# Bacteriophage-host interactions as a platform to establish the role of phages in modulating the microbial composition of fermented foods

**DOI:** 10.20517/mrr.2021.04

**Published:** 2022-01-12

**Authors:** Kelsey White, Jun-Hyeok Yu, Giovanni Eraclio, Fabio Dal Bello, Arjen Nauta, Jennifer Mahony, Douwe van Sinderen

**Affiliations:** ^1^School of Microbiology & APC Microbiome Ireland, University College Cork, Cork T12 YT20, Ireland.; ^2^Sacco Srl, Cadorago (Co), Milan 20133, Italy.; ^3^FrieslandCampina, Amersfoort 3800 BN, The Netherlands.; ^#^Authors contributed equally.

**Keywords:** Bacteriophage, phageome, prophage, fermented foods, metagenome, receptor, anti-phage activity

## Abstract

Food fermentation relies on the activity of robust starter cultures, which are commonly comprised of lactic acid bacteria such as *Lactococcus *and *Streptococcus thermophilus*. While bacteriophage infection represents a persistent threat that may cause slowed or failed fermentations, their beneficial role in fermentations is also being appreciated. In order to develop robust starter cultures, it is important to understand how phages interact with and modulate the compositional landscape of these complex microbial communities. Both culture-dependent and -independent methods have been instrumental in defining individual phage-host interactions of many lactic acid bacteria (LAB). This knowledge needs to be integrated and expanded to obtain a full understanding of the overall complexity of such interactions pertinent to fermented foods through a combination of culturomics, metagenomics, and phageomics. With such knowledge, it is believed that factory-specific detection and monitoring systems may be developed to ensure robust and reliable fermentation practices. In this review, we explore/discuss phage-host interactions of LAB, the role of both virulent and temperate phages on the microbial composition, and the current knowledge of phageomes of fermented foods.

## INTRODUCTION

It is estimated that the deliberate fermentation of foods and beverages as a means to extend their shelf-life has been practised for almost 13,000 years^[1]^. Early food fermentations were introduced at this time on every continent and fermentation substrates encompassed regionally and seasonally available raw materials, including animal milk, meats, fish, cereals, vegetables, legumes, seeds, roots, and fruits^[2,3]^. While the earliest fermentations were spontaneous and prone to quality variation and failure, modern industry has adapted these fermentations to facilitate large scale productions with highly reproducible outcomes. Consequently, an abundance of fermented foods is manufactured globally, and their combined commercial value is estimated at 30 billion dollars per annum^[4]^. In addition to the preservation of various products, fermentation can impart desirable organoleptic properties (i.e., textures, flavours, appearances, *etc.*) to the final product^[5]^. Furthermore, the contributions of fermented foods to satisfy human dietary requirements and support health are highlighted by the beneficial effects of live microorganisms (probiotics) and soluble factors released from inactivated probiotics (post-biotics), as well as by a wide range of macro- and micro-nutrients^[6-8]^.

Food fermentation processes encompass biochemical transformations of various organic substrates to metabolites (i.e., lactic acid, alcohol, free fatty acids, ammonia, *etc.*) through the enzymatic activities of specific microorganisms^[7]^. One specific group of microorganisms is intrinsically associated with food fermentations, i.e., the lactic acid bacteria (LAB), which include genera such as *Lactococcus, Leuconostoc*, *Pediococcus*, *Streptococcus*, *Enterococcus*, *Carnobacterium*, *Alkalibacterium*, *Lactobacillus*, *Lacticaseibacillus*, *Lactiplantibacillus*, *Levilactobacillus*, *Ligilactobacillus*, *Limosilactobacillus* and *Weissella*^[3]^. Depending on their role, LAB can be divided into two groups: (1) starter LAB, which is primarily responsible for acidification; and (2) non-starter LAB (NSLAB) that typically play a role in the ripening and maturation process^[9]^. Since starter LAB initiate and control the overall fermentation process by reducing the pH of the raw starting material, the selection of robust and technologically appropriate starter strains is critical to obtain high-quality products^[10]^.

Traditional and artisanal production systems commonly rely on the indigenous microbiota of the substrates or production vessels^[11]^. These “natural” starters are predicted to continuously evolve, and fermentation may be achieved through a process termed “back-slopping”, where a portion of the previous fermentate is used to initiate the next round of fermentation^[12]^. This back-slopping approach is used in many regional production systems and utilises mixed strain starter (MSS) cultures whose composition is undefined [Table 1]. Production systems that apply undefined MSS range from farmhouse, small-scale production systems to large-scale, modern industrial fermentations. In meat fermentations, members of the *Lactobacillales* and pediococci tend to be most abundantly present, while *Staphylococcus carnosus* and *Micrococcus *spp. may also be present. In vegetable fermentations, *Lactiplantibacillus plantarum *(among other *Lactobacillales*), *Weisella *and *Pediococcus *spp. are highly abundant^[13]^. In dairy fermentations, the microbiota is dependent on the fermentation temperature, being either mesophilic or thermophilic. Mesophilic fermentations typically incorporate strains of *Lactococcus lactis *or *Lactococcus cremoris *and, in some cases *Leuconostoc *and *Lactobacillales*. Thermophilic dairy fermentations typically include strains of *S. thermophilus* and members of the *Lactobacillales*. Artisanal production systems may also incorporate additional organisms (e.g., enterococci) through the application of unpasteurised milk or traditional production vessels. Undefined artisanal cultures that have been preserved (in place of the back-slopping approach) may be directly applied to initiate the fermentation, in which case the culture is not continuously evolving as the starter culture is derived from a master stock. From these undefined artisanal and mixed starter cultures, individual strains have been selectively isolated based on their desirable technological properties. These individual strains may be used in industrial fermentations in so-called “defined strain starter” (DSS) cultures in which a small number of strains (typically two to six strains) are combined to achieve products with specific organoleptic properties [Table 1]^[14]^. Such DSS cultures are widely applied in the production of Cheddar-style cheeses. Furthermore, individually isolated and characterised strains may be applied as adjunct cultures to (1) ensure rapid acidification of the substrate and/or (2) support cheese ripening^[15,16]^.

**Table 1:** Typical composition and applications of different starter cultures

**Starter type**	**Typical composition**	**Applications**
Artisanal	Undefined composition of mesophilic or thermophilic bacteria, occasionally fungi	Regional products, e.g., fermented meats, vegetable fermentation, Caciocavallo, Pecorino, Vastedda cheeses
Mixed strain starter	Undefined composition of mesophilic or thermophilic bacteria	Fermented products, e.g., Gouda, Edam cheeses
Defined strain starter	Defined composition of typically, mesophilic bacteria, e.g., *Lactococcus lactis *or *cremoris*	Cheddar style cheeses
Non-starter LAB	Typically, mesophilic LABs, including *Lactobacillales *genera	Spontaneously present and may contribute to flavour but may also cause product inconsistencies

LABs: Lactic acid bacteria.

### Bacteriophages in fermented food

Phages are viruses that specifically infect bacteria, and are considered the most abundant biological entities on earth that co-habit any ecological niche where bacteria exist^[17]^. Since DSS cultures comprise a small number of strains, bacteriophage (phage) infection of one or more strains in the culture may have a catastrophic impact on the production regime and the final properties of the product^[18]^. In contrast, phage infection of strains within complex mixed starter cultures is less likely to impact severely on the production regime, though product inconsistencies may occur. In the context of fermented vegetables, phage predation has been associated with the progression of the LAB landscape and which is central to the development of the flavour and aroma profile of these products^[13]^. LAB-infecting phages represent one of the most significant challenges in the dairy fermentation industry, with infection of starter cultures being a common cause of production delays or even complete arrest of the fermentation process^[19]^. Among phages of LAB, those that infect *Lactococcus lactis*, *Lactococcus cremoris* and *Streptococcus thermophilus* have been studied most extensively and will, therefore, be the primary focus of this review^[11,20]^.

All currently known LAB phages belong to the *Caudovirales* order of tailed phages, which possess a double-stranded DNA-containing capsid^[17]^. Lactococcal phages are classified based on their morphology and nucleotide homology into 10 taxonomic groups, while an additional novel isolate called phage Nocturne116 was described recently^[21,22]^. Among the described lactococcal phage groups, three are most frequently encountered in industrial fermentations: the P335, Skunaviruses (formerly called 936) and the Ceduoviruses (formerly called c2) groups. Members of the *Skunavirus* and *Ceduovirus* groups are highly problematic groups as they are virulent phages, whereas members of the P335 group may be virulent or temperate^[23,24]^. While certain genomic regions of the Skunaviruses and Ceduoviruses are highly conserved (within a phage species), specific regions of diversification have also been reported, including those that encode host-binding domains within their neck passage structure and tail proteins^[25,26]^. Conversely, the P335 phages are rather heterogenous and appear to possess a mosaic genome structure, likely as a result of genomic recombination between related phages^[27,28]^. Temperate phages may exist in the virulent state, or they may integrate their chromosomes into that of the host bacterial cell, in which state they are termed prophages. The decision between the lytic and lysogenic lifestyles of temperate phages is assumed to be dictated by the availability of host cells and environmental conditions/cues. While temperate phages can exist in a dormant, prophage state in the genomes of their host starter strains without adverse effects on fermentation, they present an ever-present risk to the fermentation process should they revert to the virulent state^[29,30]^.

*S. thermophilus* phages are problematic in thermophilic production systems, and several studies have reported their global prevalence in and the corresponding impact on industrial food fermentations^[31-33]^. *S. thermophilus* phages were originally classified into two major groups based on their structural protein content and DNA packaging mechanisms^[34]^. These two phage groups were termed the *cos* and *pac* groups, and have recently been renamed the Moineauviruses and Brussowviruses, respectively^[35]^. In 2011, the novel phage isolate 5093 was identified as a representative of a new phage group, followed by the identification of two additional novel phage groups, i.e., the 987 and P738 groups^[36-38]^. While the Moineau- and Brussowviruses continue to be the most prevalent dairy streptococcal phage groups (69% and 29%, respectively), the emergence of novel phage groups, possibly through recombination with phages of other streptococcal species or LAB such as *Lactococcus* spp., highlights the ongoing need for phage monitoring. Moineauviruses are virulent phages, while members of the *Brussowvirus *group may be virulent or temperate^[20]^. While the incidence of prophage induction in *S. thermophilus *appears to be rather low (2%), many apparently complete prophages or their remnants are present in their chromosomes, facilitating genomic plasticity of their phages contributing to the mosaicism and diversification^[33,34,39-41]^. Furthermore, it is noteworthy that recombination-driven genetic shuffling and exchange events of functional modules have been observed between lytic phages^[32]^.

## PHAGE-HOST INTERACTIONS

To understand how phages influence the overall microbial community composition in fermented foods, it is important to consider the diversity and basis of phage-host interactions occurring in these communities. Phage infection commences with the recognition of, and adsorption to, a receptor on the host cell surface, often involving an initial reversible binding followed by irreversible binding and associated commitment to infection^[42]^. The initial reversible binding step is facilitated by the phage-encoded anti-receptor, which typically comprises one or more receptor binding proteins (RBPs) located at the distal end of the phage tail, commonly supported by auxiliary host binding proteins^[26,43]^. RBPs may recognise saccharidic, teichoic acid, and/or proteinaceous receptors [Table 2]. Considerable research has been undertaken to understand phage-LAB host interactions, which has rendered them a paradigm for a diverse range of microorganisms, but particularly Gram-positive bacteria that are infected by tailed phages.

**Table 2:** Defined and putative phage receptors of LAB

**Host**	**Phage Group**	**Representative phage**	**Receptor type**	**Host receptor**	**Ref.**
*Lactococcus* spp.	*Skunavirus*	bIL170	Saccharidic	CWPS	[44]
	P335	Tuc2009			[45]
	1358	1358			[46]
	949	949			[47]
	P087	P087			[48]
	1706	CHPC971			[49]
	*Ceduovirus*	c2	Proteinaceous	PIP	[50,51]
		bIL67		YjaE	[52]
*Streptococcus thermophilus*	*Moineauvirus*	CHPC1014	Saccharidic	RGP	[53]
	*Brussowvirus*	CHPC951		RGP	[53]
	5093	5093		*Unknown*	[54,55]
	987	9871		EPS	[56]
*Lactobacillus delbrueckii*		LL-H	Teichoic acid	LTAs	[57]
*Leuconostoc mesenteroides*	Ia	P842	*Unknown*		[58]
	Ib	ΦLN25			
*Leuconostoc pseudomesenteroides*	IIa	P839	*Unknown*		[58]
	IIb	P791			

EPS: Exopolysaccharide; RGP: rhamnose-glucose polysaccharide; CWPS: cell wall polysaccharide structures; PIP: phage infection protein; LTAs: lipoteichoic acids.

### Host receptors

#### Saccharidic receptors

The interactions between lactococcal and streptococcal phages and their cognate host have been the focus of intense research scrutiny over the past three decades^[56,59-61]^. The majority of streptococcal and lactococcal phages recognise saccharidic receptors: exopolysaccharide (EPS) or rhamnose-glucose polysaccharide (RGP), and cell wall polysaccharide structures (CWPS), respectively^[56,62]^. Dupont *et al.*^[44]^ identified the role of the *cwps *gene cluster in lactococcal phage adsorption by means of random insertional mutagenesis of two *Lactococcus *strains (*L. lactis *IL1403 and Wg2). Through this approach, bacteriophage insensitive mutant derivatives exhibiting reduced phage adsorption capabilities were isolated. The *cwps *gene clusters of lactococci typically encompass a DNA region of 25-30 kbp^[62-65]^. Due to the ever-increasing number of available genome sequences, it has been possible to interrogate the functions and genetic diversity of these clusters^[62-65]^. The *cwps* gene cluster is responsible for the biosynthesis of two CWPS elements: the peptidoglycan-embedded rhamnan (whose biosynthetic machinery is encoded by the 5’ portion of the *cwps* cluster) and surface-exposed polysaccharide pellicle (PSP; the biosynthesis of which is performed by enzymes that are encoded by the 3’ portion of the *cwps* cluster). Of the 11 distinct groups of lactococcal phages, *Skunavirus*, P335, 1358, 949, P087, and 1706 phages have been demonstrated to utilise saccharidic host receptors^[44-49,63,66]^. These lactococcal phages specifically bind to the PSP component of the CWPS on the host cell surface, mediated by the phage RBP. The genetic diversity of this gene cluster among lactococcal strains, particularly in the 3’ region corresponding to distinct glycosyltransferase-encoding genes, is responsible for the biochemical diversity observed in the PSP between strains. Lactococcal strains can, therefore, be grouped based on differences in the genetic composition of the gene cluster responsible for CWPS biosynthesis^[62-65]^. There are currently four defined *cwps *genotypes designated by type A-D, with C types further subdivided into eight subtypes (C_1_-C_8_)^[62,64]^. A recent comparative analysis encompassing over 400 lactococcal sequences (including an industrial strain collection) also indicates the presence of several additional *cwps* types (A-H) and subtypes, suggestive of a continually evolving genetic composition of this gene cluster^[67]^. Furthermore, the different genotypes defined among these loci correspond to distinct CWPS chemical structures, thereby facilitating the prediction of structural features of lactococcal CWPS, including the number and order of component monosaccharides in the PSP, the likelihood of an oligosaccharide or polymeric (PSP) side chain and the presence of chemical modifications of the component monosaccharides^[62]^.

Four of the five streptococcal phage groups (all except the newest P738 group for which this has not yet been studied) utilise saccharidic receptors on *S. thermophilus *cell surfaces^[38,68,69]^. The polysaccharide structures produced by *S. thermophilus *are either the loosely cell wall-associated EPS or the more tightly cell wall-bound CWPS. The gene clusters responsible for the biosynthesis of *S. thermophilus *EPS and CWPS are *eps* and *rgp*, respectively. *Moineauvirus* and *Brussowvirus* phage RBPs have been determined to recognise the host RGP^[54,55]^, whereas the RBP of the more recently described 987 group phages was found to target EPS structures^[36,38]^. Based on the sequences of 167 *S. thermophilus *strains (many of which are industrial strains), it has recently been proposed that there are three RGP groups (A-C), with 18 further subtypes^[67]^. This would represent an expansion on and reclassification of the previously proposed *rgp* grouping into types A through to E^[70]^. There are also six defined *eps *types (A-F)^[70]^ (for a detailed review of the genetic diversity of these loci see^[67]^). The genetic diversity of the gene clusters (and subsequent biochemical diversity) responsible for the biosynthesis of these cell wall polysaccharide structures accounts, at least in part, for the highly specific nature of LAB phage-host interactions, i.e., the narrow host range observed for many LAB phages.

#### Proteinaceous receptors

While many LAB phages use saccharidic receptors to infect their bacterial hosts, lactococcal Ceduoviruses have been found to bind reversibly to a saccharidic moiety and irreversibly to a proteinaceous receptor. Based on comparative genomics and host specificity, Ceduoviruses are classified into two subgroups: the c2-type and bIL67-type phages^[71]^. The proteinaceous receptor these phages bind to is either the phage infection protein (PIP; in the case of c2-type phages) or YjaE (for bIL67-type phages)^[61]^. The genes encoding these proteins are ubiquitous and well-conserved in lactococcal strains. Consequently, the host range of Ceduoviruses tends to be much broader than those of Skunaviruses, among other lactococcal phages^[72]^. However, some Ceduoviruses have been found to have a preference for certain CWPS types, demonstrating how this initial, reversible step of infection may still be crucial and subsequently restrict a phage’s potential host range^[27]^.

#### Teichoic acid receptors

Teichoic acids are phosphodiester-linked co-polymers (of glycerol- or ribitol-phosphate and carbohydrates) that represent a ubiquitous component of the cell envelope of Gram-positive bacteria. There are two groups of teichoic acids: lipoteichoic acids (LTAs) and wall teichoic acids (WTAs). A number of *Siphoviridae *phages use WTAs or LTAs as an initial receptor through reversible binding during phage infection. Phages infecting various *Bacillus*, *Listeria*, and *Staphylococcus *species use WTAs as receptors, as WTAs are the most abundant surface molecule in the Gram-positive bacterial order *Bacillales*^[42]^. Although limited information exists pertaining to the host receptors of *Lactobacillus *phages, it has been demonstrated that phage LL-H which infects *Lactobacillus delbrueckii *ssp. *lactis *employs LTAs for the purpose of host recognition^[57]^.

### Phage anti-receptors

**Figure 1:**
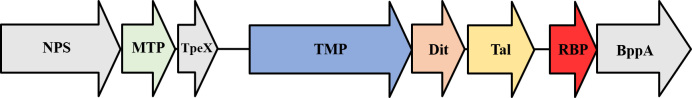
Schematic representation of genes commonly present in the tail morphogenesis region of lambda-like *Siphoviridae *recognising saccharidic receptors. The late-expressed genes that are commonly shared by these phages and make up the tail include the: major tail protein (MTP; green), tape measure protein (TMP; blue), distal tail protein (Dit; orange), tail-associated lysin (Tal; yellow), and receptor binding protein (RBP; red). Additional carbohydrate-binding modules (CBMs) found in auxiliary binding proteins (grey) have been identified and characterised in certain phages, such as the: neck passage structure (NPS), major tail extension protein (TpeX), and accessory baseplate protein (BppA). It is noteworthy that in some cases (e.g., certain P335 lactococcal phages), the NPS-encoding gene is located downstream of the RBP-encoding gene.

Despite the genetic diversity exhibited by siphophages, the genome architecture and synteny of the functional module responsible for tail morphogenesis is well conserved, incorporating the following functions (in a 5’ to 3’ direction): the tail tape measure protein, the distal tail protein (Dit), and the tail-associated lysin (Tal). This region is typically followed by genes encoding the baseplate proteins, including the RBP, with auxiliary binding proteins in many cases [Figure 1]. The RBP-encoding genes of many of these phages were initially identified through comparative genome analysis, and representative RBPs have been well characterised in both lactococcal and dairy streptococcal phages^[70,71,73,74]^. Owing to the vast number of LAB phages whose genomes have since been sequenced, knowledge pertaining to phage anti-receptors, as represented by both RBPs and auxiliary binding proteins, has been substantially expanded and is now well defined for many lactococcal and streptococcal phages, whereas those of other LAB genera still requires considerable research attention.

#### Recognition of saccharidic receptors

The RBP of Skunaviruses was first identified in lactococcal phages sk1 and bIL170 using an *in silico* approach, and functionally assigned following isolation of recombinant phages encoding chimeric RBPs^[74]^. The 3-dimensional structure of the RBP of lactococcal phage p2 was shown to represent a homotrimer of three domains: the shoulders, neck, and head^[75]^. The head domain (C-terminal end) encompasses the actual receptor binding activity^[76]^. For Skunaviruses, comparative analysis of C-terminal sequences from various RBPs facilitated a phylogenetic grouping^[63]^. Currently, five *rbp *genotypes are defined that correlate well to the specific *cwps* genotype of their corresponding host(s)^[65]^. In addition to the receptor binding ability of the RBP C-terminal or head domain, a lactococcal virion may possess auxiliary CBM decorations on the Dit, major tail protein, and neck passage structure (NPS) that may all contribute to host cell binding. These CBMs facilitate specific host binding and indeed exhibit the same host specificity of its corresponding RBP albeit, in some cases, with an apparently reduced affinity relative to this RBP^[26]^.

The RBP of lactococcal P335 phages was identified for phages Tuc2009 and TP901-1 where it was observed to form part of a multi-protein complex called a baseplate and in which the RBP was identified as the lower baseplate protein (BppL)^[66]^. Certain P335 group phages recognise CWPS structures, although no direct correlation between RBP subgroups and host CWPS has been determined to date, likely due to the heterogeneity of the P335 phage group^[23,64]^. However, it has been suggested that P335 phages may have a preference for *cwps* type A strains over type C or B strains based on a study incorporating a selection of 39 lactococcal strains and 17 P335 phages isolated from whey samples derived from cheese production facilities across multiple continents^[23]^.

Similar to lactococcal phages, a number of phage-tail proteins are involved in the host binding process among *S. thermophilus *phages. The Tal was originally thought to be the protein primarily responsible for host specificity, among additional genetic determinants yet to be identified^[77]^. The bona fide RBP was later identified downstream of the Tal-encoding gene, as well as other genes specifying auxiliary CBMs that appear to facilitate host binding^[55]^. The functionality of the individual CBMs as well as the distal tail structure of streptococcal phage STP1 (*Moineauvirus*) was also determined^[55]^. The specific saccharidic host receptor of 5093 phages is yet to be confirmed. However, an esterase-like domain is present in the C-terminal end of a putative RBP, being consistent with the finding that saccharidic components on the *S. thermophilus* cell wall (such as the EPS and CWPS) incorporate phosphodiester-linked carbohydrate moieties^[54,55]^.

Host adhesion is not limited to the RBP and is often aided by a number of auxiliary binding proteins found to contain additional CBMs in many lactococcal and streptococcal phages, including the: NPS, TpeX, BppA, and Dit. In certain Skunaviruses and P335 phages, the NPS forms a collar-whisker complex attached to the phage portal and contains a CBM involved in, but not essential to, host binding^[78]^. In addition, a TpeX has been identified in certain Skunaviruses and results in the presence of additional decorations along the tail. Through fluorescence binding assays, the CBMs of the NPS and TpeX have been determined to exhibit the same host specificity as the RBP (though with inferior affinity when compared to that of the RBP) and bind favourably towards the ends of the cell where cell division occurs^[26]^. Certain P335 phages also have an additional CBM located in an auxiliary binding protein known as the BppA^[45,60]^. Most lactococcal and streptococcal phages encode a Dit, which is either classified as classical or evolved. Evolved Dits are longer and contain an insertion of an additional CBM, which has been found to exhibit the same host specificity as the RBP^[23,55,79]^. 5093 and 987 phage Dits are classical and do not incorporate CBM insertions. Lactococcal and streptococcal phages tend to possess a variety of CBMs (in addition to the RBP) in somewhat conserved combinations, and very rarely contain none or all possible auxiliary CBMs^[26]^. Beyond lactococci and dairy streptococci, limited studies have focused on the identification of the receptor moiety of LAB phages; however, it seems likely that many employ a saccaharidic receptors given their narrow host ranges. The phages of *Leuconostoc*, for example, are divided into two major groups that coincide with their host bacterial species, i.e., *Leuconostoc mesenteroides *and *Leuconostoc pseudomesenteroides*^[80]^. The receptor binding protein of these phages has been identified through the generation of phages harbouring chimeric RBPs and the host range and morphological alterations attributed to the “swapped” RBP domains.

#### Recognition of proteinaceous receptors

As mentioned above, *Ceduovirus *members are the only lactococcal phage members known to recognise and bind irreversibly to a proteinaceous receptor^[50]^. Relative to other lactococcal phages, there is limited information regarding the exact phage-encoded protein(s) responsible for host binding among members of the *Ceduovirus *group. While overall, there is limited sequence divergence among Ceduoviruses, a cluster of three genes displays reduced sequence similarity among members of this group. This cluster, which contains three late-expressed genes: *l14*, *l15*, and *l16 *in phage c2 and its equivalents ORF34, ORF35, and ORF36 in bIL67, has been suggested to be responsible for host recognition in *Ceduovirus *phages c2 and bIL67^[71]^. This three-gene region is proposed to encode structural proteins responsible for binding to the host Pip or YjaE, however, the exact function of these genes is yet to be elucidated^[61,71]^. The non-LAB *Bacillus subtilis*-infecting phage SPP1 also uses a proteinaceous receptor for infection and has been thoroughly studied. SPP1, like *Ceduovirus *phages, first binds reversibly to a saccharidic receptor and then irreversibly to a cell surface-located proteinaceous receptor, YueB, which bears similarity to the lactococcal PIP^[81,82]^. The distal tail complex of SPP1 is well described and is structurally similar to the tail of lactococcal phages. Due to these similarities, SPP1 (despite infecting a non-LAB host) is a model for tailed phages that employ proteinaceous receptors^[50]^.

Although much research has been dedicated in recent years to defining phage-host interactions between *Siphoviridae* and their Gram-positive hosts, in particular LAB, additional insights are required to fully understand these interactions at the molecular level. Also, due to the conserved nature of the tail structure of many Gram-positive host-infecting* Siphoviridae *phages, knowledge gained from understanding phage-host interactions in one group of bacteria (such as LAB) may be superimposed to better understand phage-host interactions involving other bacterial groups. One of these groups being *Enterococcus *strains, which enter the fermented food process (particularly that of cheese) either in the raw materials (such as milk) or at other stages of the manufacturing process. Little is known about the phages infecting these hosts in fermented foods, but they still may play a role in the microbial composition of these foods and should be further studied^[83]^. In addition, with respect to phage-host interactions within a microbial community, knowledge gained from understanding individual interactions may be applied and expanded to understand the network of phage-host interactions within more complex microbial communities across various environments.

## ROLE OF VIRULENT PHAGES IN MODULATING MICROBIAL COMPOSITION OF FOOD

In industrial settings, virulent phages can represent a double-edged sword, depending on the environment and their host. For example, phages have been used as a non-chemical biocontrol tool to eradicate contaminating pathogenic species such as *Listeria monocytogenes* from many food products^[84]^. However, in the overall context of fermented food production, the presence of virulent phages that infect starter cultures is undesirable as they can cause slow or failed fermentation, with significant associated economic losses. In dairy fermentations, the impact of virulent phages on the fermentation process differs depending on the starter culture format, i.e., defined or undefined starter cultures, as well as the scale of fermentation, i.e., industrial scale *vs.* artisanal production systems.

Erkus *et al.*^[85]^ demonstrated that phage-sensitive strains are replaced by phage-insensitive strains of the same lineage, allowing for continued fermentation when using the complex Gouda cheese starter culture Ur. The composition of the original starter culture was dissected using culture-dependent and independent methods, allowing for community monitoring using genetic lineage-specific qPCR. The culture was able to maintain the relative composition of the different lineages, despite phage (originating from the original starter culture) pressure on individual strains. This was determined to be due to heterogeneity of the culture and, more specifically, variations in phage sensitivity of strains within and between lineages^[85]^.

Phage attack can have a significant effect on both DSS and undefined starter cultures, although the impact and means to mitigate phage infection may vary. In DSS cultures, the specific composition, phenotypic, and behavioural characteristics of each strain that compromise the culture are known. If one or more of the strains in the DSS culture becomes infected by virulent phages, such strains may be readily replaced with phage-unrelated strains or phage-insensitive derivatives (possessing the same technological characteristics). This process ensures successful fermentation and product consistency/quality. Conversely, in undefined starter cultures, if the acidification of milk continues despite phage attack, the organoleptic properties or quality of the final product may be negatively impacted^[86]^. Phages infecting NSLABs present in the starter culture do not tend to impact acidification because these strains are typically utilised in fermentation specifically for the organoleptic properties they impart*.* In many cases, these inconsistencies are not observed until grading of the product occurs, at which point flavour, aroma, eye formation and surface ripening are evaluated as appropriate to the specific product. Such product inconsistencies are difficult to identify during the production process and can be costly to food producers through product down-grading.

In addition to the negative impacts of virulent phage predation on microbial communities in fermentation, lytic phage also plays an important role in the composition and evolution of starter cultures by driving the genetic diversity of bacterial strains. For example, simple blends composed of representative strains from different genetic lineages (with varying phage sensitivity profiles) were created from the undefined culture Ur^[18]^. The relative abundance of the genetic lineages was monitored across sequential rounds of propagation in the absence or presence of phage pressure. Using this more defined version of a complex starter culture, the genetic lineages did not remain stable during sequential rounds of phage attack. However, phage-resistant variants eventually arose from phage-sensitive genetic lineages, after which the cultures stabilised to the same relative composition as control blends without the presence of phage^[18]^. This study demonstrates how phages are key contributors driving the diversity among bacterial strains.

Traditional and artisanal cheeses are produced based on starter cultures that typically consist of autochthonous bacteria already present in the fermentation materials (such as raw milk) or environment (e.g., fermentation vats). These cheeses are mostly produced using traditional production methods with fewer chemical and physical hurdles for phages to overcome, thereby allowing phage populations to emerge that are different when compared to those of modern fermentation facilities. For example, in Sicilian artisanal cheese facilities, it was shown that 16 of the 18 phages isolated from associated cheese whey and rennet samples belong to the 949 and P087 lactococcal group phages, which are rarely isolated from whey samples obtained from modern, large-scale cheese factories^[11]^. These phages are much more heat sensitive compared to the other more dominant lactococcal phages (such as Skunaviruses) and appear to thrive in this traditional fermentation environment due to the lack of pasteurisation^[11]^. While phages are a driving force of bacterial evolution, they are also continuously adapting and evolving in response to their hosts when the latter acquire resistance. Phages may mediate the transfer of genetic material via transduction (transfer of bacterial genetic material that has been packaged into the capsid of the phage), although this typically occurs at low frequencies. In contrast, temperate phages have the potential to contribute significantly to the transfer of genetic material from one strain to another and ultimately contribute to the evolution of a given bacterial species. In the ensuing section, we will explore the impact of temperate phages on starter bacterial species and culture composition in food fermentations.

## IMPACT OF TEMPERATE PHAGES ON STARTER CULTURE COMPOSITION

Starter culture bacteria, including most *Lactococcus *species and *Lacticaseibacillus rhamnosus* and *Lactiplantibacillus plantarum* (vegetable fermentations) typically harbour one or more prophages in their genomes^[87-89]^. Prophage-mediated lysis of the culture may be considered an ambivalent phenomenon since it can confer both positive and negative effects on the associated fermentation product, i.e., culture lysis can cause incomplete/delayed acidification, while it may also cause the release of intracellular enzymes associated with flavour development. Also, interactions between host bacteria and prophages generate significant changes in bacterial chromosomes through the adoption and rearrangement of the functional module from prophages, resulting in the evolution of host bacteria as well as phages^[28]^.

### Induction of prophage(s) from starter strains

Since the phenomenon of lysogeny in LAB was first reported in 1949 by Reiter^[90]^, the prevalence of lysogens in starter culture strains has been highlighted in many studies^[87,91-93]^. For example, Terzaghi and Sandine^[91] ^(1981) showed that all 45 tested lactococcal strains suffered from growth cessation and/or lysis following UV treatment along with the frequently observed release of phage particles. Regarding *S. thermophilus*, intact prophages or, more commonly, phage remnants (present as incomplete prophage genomes) are frequently observed, indicating that most *S. thermophilus* strains have been challenged by lysogenic phages^[40]^. Furthermore, various applications such as flow cytometry (FCM) have recently been developed to overcome the limitations of plaque-based methods, which are time-consuming and limited to infectious phages. Using FCM, detection of induced prophages is more precise without false-negative results^[91,94-96]^. These findings highlighted the risk of prophage-carrying starter strains and led to a move away from traditional mixed starter fermentations for certain applications (such as Cheddar cheese production) where a consistent product profile is required.

In contrast, Kelleher *et al*.^[87]^ (2018) showed that only four out of 24 evaluated lactococcal strains, apparently possessing one or more intact prophages, formed intact phage particles following MitC (mitomycin C) exposure. During commercial fermentations, strains may be subjected to various stressful conditions such as high salt concentration, high (or low) temperatures, or extended exposure to low pH, though this does not seem to affect prophage stability, suggesting that prophage induction under production conditions does not appear to occur often^[97]^. In addition, lysis of starter culture cells during the ripening process is regarded as beneficial for flavour formation, as long as the acidification process is unaffected^[98]^. The release of intracellular enzymes from lysed cells and accessibility to substrates (i.e., casein and its peptide and amino acid breakdown products) promotes flavour development. Autolysis (and in some cases, prophage-mediated lysis) may be regarded as a critical step to achieving high-quality products. The correlation between “leaky” prophages and bacterial autolysis has been investigated with various starter strains. In particular, Husson-Kao *et al*.^[99]^ (2000) proposed that the observed autolytic properties of a particular *S. thermophilus* strain are associated with the constitutive expression of phage genes, performing auxiliary roles for cell lysis. Furthermore, O’Sullivan *et al.*^[100]^ (2000) proposed that the autolytic behaviour of lactococcal strains is associated with the presence of specific phage genes in the bacterial chromosomes, i.e., lysin- or holin-encoding genes. Notably, these lactococcal strains showed autolytic behaviour under the Cheddar cheese cooking temperature (38-40 °C), indicating the lysogenised starter strains can be used deliberately to improve the quality of products. These findings not only countered previous studies that present the undesirable aspects of prophages, but also highlighted the possibilities of prophages to be utilised in a positive manner for fermentation industries.

### Interactions between genomes of bacteria and prophages

**Figure 2:**
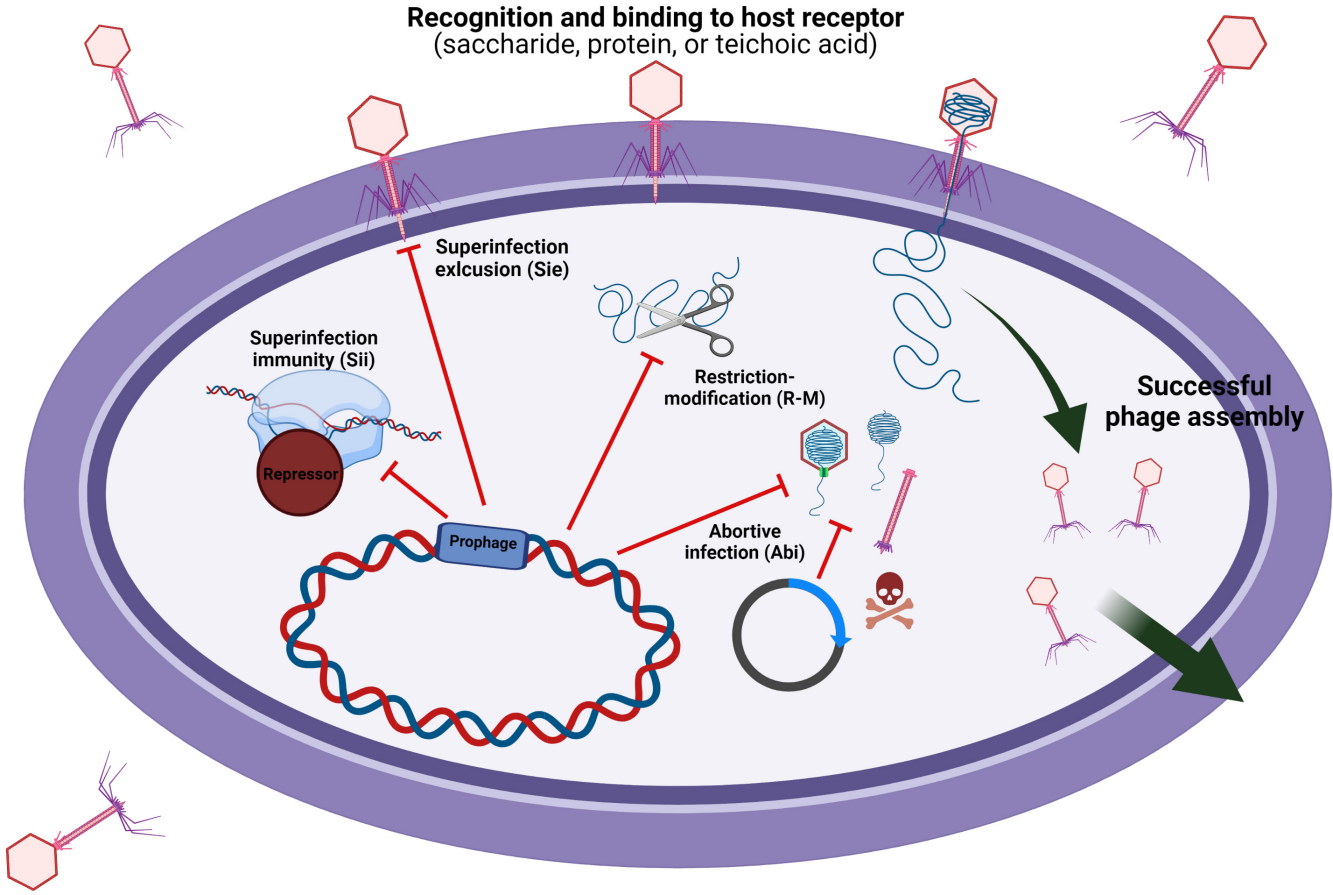
Schematic representation of commonly occurring phage-host interactions. After a phage recognises and binds to a specific host receptor, a number of plasmid-, chromosomal-, and/or prophage-derived anti-phage systems may impede successful phage proliferation, such as: Sii, Sie, R-M, or Abi systems. Created with BioRender.com.

Even if prophages are dormant without the risk of excision, the presence of prophages may significantly influence host metabolism and genetic recombination. Many studies have highlighted the role of prophages as a reservoir of genetic variations, which facilitates the evolution of host bacteria resulting from the acquisition of prophage-derived anti-phage systems. These phage-derived defence mechanisms include adsorption inhibition, abortive infection (Abi), restriction-modification (R-M), or DNA injection blocking in *L. lactis* and *S. thermophilus*^[20,28] ^[Figure 2]. Ladero *et al*.^[101]^ (1998) reported the superinfection immunity (Sii) displaying homo-immunity against superinfecting phage. This defence system blocks transcription of the lytic genes of homologous phages by expression of the repressor of their genetic switch following phage-genome entry into the cytoplasm. The repressor gene from *Lacticaseibacillus* phage A2 was identified, and the complete inhibition of phage infection against identical phage under the expression of the gene was observed when the phage genome was integrated into the bacterial host chromosome.

Another phage defence mechanism termed superinfection exclusion (Sie) typically presents as a membrane-associated protein encoded by a gene in the lysogeny module. It is believed to provide resistance against heterogenous phages and block the initiation of superinfection by preventing DNA injection. The Sie_2009_ system, encoded by lactococcal host strain UC509 harbouring temperate phage Tuc2009, is the best-understood phage exclusion system in LAB. Though its precise mode of action still remains unknown, its expression was found to cause DNA injection blocking^[102]^. However, lactococcal strains harbouring the *sie_2009_* gene are still sensitive against many tested phages, suggesting that full anti-phage activity requires high expression^[103]^. Furthermore, Ruiz-Cruz *et al*.^[104]^ (2020) showed the prophage-carriage in *Lactococcus* provided resistance against various heterogenous phage groups, including Skunaviruses (or 936), P087, 949, as well as P335 phages.

Abi systems prevent phage proliferation through the interference of an essential cellular activity such as DNA replication, transcription or protein synthesis before the completion of the phage infection cycle, resulting in host death and the production of few/no progeny phage particles^[105]^. Abi systems sense phage infection by the transcriptional and translational material of phages or their replication, before activating the cell-killing module of the Abi system. Various chromosomally- and plasmid-encoded Abi types (A-Z) have been studied, while Abi-encoding genes have also been identified as associated with prophages^[106]^. Kelleher *et al.*^[87]^ (2018) reported that nine out of 30 assessed lactococcal strains possess one or more known Abi-encoding genes on their prophages. In addition, prophage-encoded Abi systems were also identified on genomes of *Levilactobacillus brevis*, *Lacticaseibacillus paracasei*, *Limosilactobacillus fermentum*, *Lacticaseibacillus rhamnosus*, *Lactiplantibacillus plantarum*, and *Lactobacillus gasseri*^[107]^. Furthermore, the prophage-derived AbiL_124_ system exhibiting specific activity against phages infecting *Latilactobacillus brevis* and *Lactococcus lactis* demonstrated the potential of Abi systems to be used to generate novel phage-resistant starter strains.

R-M systems protect host bacteria from the invasion of foreign DNA, such as phage infection, by cleaving invading DNA at specific sequences, which are protected in the resident DNA by methylation (except for Type IV system restricting incoming methylated DNA)^[108]^. Several lactococcal prophages were determined to harbour methylase genes which serve to protect the phage from endonucleolytic cleavage by host bacteria^[87,109]^. Furthermore, prophages encoding complete R-M systems confer protection to the host carrying the prophage, highlighting the potential symbiosis between the two entities^[110]^.

These Abi, Sie, Sii or R-M systems encoded by prophages are presumed to enhance resistance against a variety of phages, thus providing fitness benefits to the host bacteria. Nevertheless, the homologous recombination between resident prophages and secondary infecting virulent phages contribute to the evolution of phages. In particular, the loss of lysogenizing functions of prophages by genomic rearrangement with infecting virulent phage genomes may result in the appearance of obligate lytic phages with the consequence of disruption of the fermentation process^[30]^. In addition, the metabolic burden of the lysogenized phages often impacts the fitness of the host strain despite the advantage to the host^[111,112]^. These findings highlight the ambivalent traits of prophages, and the importance of continuously expanding knowledge on the interrelationships between prophages and host bacteria in food fermentations.

## PHAGEOMES OF FERMENTED FOODS

In the contemporary food fermentation industry, owing to increasing consumption and awareness of fermented foods, the establishment and implementation of a reliable and traceable manufacture system has been emphasised to achieve consistent, high-quality products. The crucial role of bacterial and/or fungal microbial communities in food fermentation processes has culminated in the generation of significant data pertaining to the microbiota of foods and food production environment and has been enhanced by recent developments in genome sequencing and meta-omics tools. In contrast, very few studies pertaining to the food production environments and their phageome, which represents the overall bacteriophage community of a given sample or environment, have been published^[113]^.

To date, the presence of phages in fermented foods has been determined using several approaches, i.e., culture-dependent methods, direct detection, and metagenomic sequencing. Several studies employing classical microbiological approaches have described the evolving microbial landscape of fermented foods such as sauerkraut, natto, fermented cucumber and wine and highlighted the role of phages in the progression/disruption of the fermentation process^[114]^. While this approach has been very informative, it relies on the ability to culture and detect all microbial components in the food. It is now understood that culture-based approaches likely represent the dominant and culturable organisms but may not represent the complete population of bacterial and/or phages that may be present. Through analysis of metagenome data sets which capture entire microbial ecosystems, some phage-associated reads have been identified, though the extraction protocol had to be optimised in order to obtain a more complete image of phage prevalence, abundance and diversity^[115]^. Consequently, more targeted extraction methods for viral nucleic acid have been developed^[116]^. Viral metagenomics (or phageomics) has clearly increased our understanding of the prevalence, abundance, dynamics, and role of phages in a number of food fermentation processes. Recently, metagenomic sequencing of viral communities in kimchi and cheese surface has highlighted the viral diversity and its correlation with bacterial diversity^[117,118]^. Especially, Jung *et al.*^[117]^ revealed that the viral communities in kimchi have a much more clear association with geographical origins than microbial communities, facilitating an in-depth understanding of fermented food ecosystems. Nevertheless, there remains an abundance of viral dark matter, which does not align with any reference virus sequences, obstructing the comprehensive understanding of the viral community. In addition, there are limited studies to date on phageome-specific extraction methods compared to the standard metagenome extractions to truly understand the potential benefits of a more targeted approach. While phageome studies of fermented foods are currently limited, it will be important to consider the sample preparation for phageome studies. The viral load and associated nucleic acid extract concentration can be low where the metagenome or direct phageome analysis approaches and identifying low abundance phages can be challenging. However, using enrichment approaches can lead to amplification of dominant members of the phageome. To overcome these challenges, qPCR systems to identify and quantify a range of phage species of the sample pre- and post-enrichment could be applied to track the changing population landscape to complement sequencing strategies. This represents an opportunity to expand and deepen the current understanding of the role, diversity and functionality of phages in food systems. To reduce the viral dark matter, not only an enrichment of viral sequences is required, but also the combinations with biological and molecular methods need to be improved.

## CONCLUSION AND FUTURE PERSPECTIVES

Phages have maintained a prominent role in modulating the microbial composition of fermented foods. The “kill-the-winner” model of phage dynamics allows for the stabilisation of complex bacterial communities^[119]^. This hypothesis states that an increase in a particular bacterial strain within a microbial community will coincide with an increase of phages that can infect that strain, thereby reducing its abundance and preventing a single strain from dominating the community. Phages, therefore, play an essential role in maintaining the diversity and stability of the complex microbial communities necessary for the production of fermented foods. Culture-based methods, and more recently, molecular and genomics-based methods have been instrumental in defining phage-host interactions among LAB. This knowledge can now be applied to better understand these interactions between phages and other lactococcal and streptococcal strains, as well as other important LAB such as lactobacilli. There is a diverse range of globally applied fermentation practices that utilise LAB, as well as other microbes. The microbes used during fermentation, whether autochthonous or introduced through starter cultures, vary depending on the geography, environmental conditions, climate, fermentation practices, and raw materials used. In addition, the demand for fermented foods is only increasing, demonstrating the importance of generating deeper insights into the microbial interactions in these communities. With an increasing demand for consistent, high-quality products, there is increasing pressure to employ robust and reliable fermentation practices. In particular, there is increasing interest in plant-based dairy alternatives, and in order to gain a better understanding of the phage-host interactions occurring in these unique environments, phageome and metagenome analysis should be utilised. Furthermore, there is an opportunity to expand current knowledge pertaining to the microbiota in fermented meat and vegetable products and to determine their contributions to metabolite production as well as product safety and quality^[120,121]^.

With the expanding use of metagenome and phageome sequencing of fermented foods, we are only now beginning to uncover the true complexity of these microbial communities. By combining both phageome and metagenomic analyses, it will be possible to gain a better understanding of the ever-evolving phage-host interactions occurring in fermentation environments. Through the combination of culture and culture-independent approaches, it is possible to achieve an in-depth, systems understanding of the genetic and functional diversity of microbial communities present in fermented foods. Analysis of CBMs in phageome data, particularly those found in phage RBPs, can be used to predict potential hosts of the phages present. A link between *Skunavirus* RBPs and host CWPS has already been defined (and may likely need to be expanded as more phages are isolated and sequenced)^[26,63,65,67]^. With continued phage-host interaction studies of LAB this predictive strategy presents a paradigm for the microbial interactions of a range of Gram-positive bacteria and their infecting tailed phages.

Constant monitoring of the microbial community is essential in order to overcome the negative impacts of phages in fermentation and ensure the production of consistent, high-quality products. To date, phageome and metagenomic sequencing in fermentation have provided just a snapshot of the diversity in specific communities. These tools need to be expanded and used to monitor shifts in populations over time and with changing environmental conditions. Metagenomics and phageomics should be used as a tool to understand the phages (both virulent and temperate) and strains that are present in a specific fermentation factory. For instance, it may be possible to monitor the microbial community by monitoring specific genes, such as those encoding phage-host receptors or strain/genetic-lineage specific genes. This can then be linked to the phageome, where problematic phages associated with a specific factory can also be monitored and detected. By utilising culturomics, metagenomics, and phageomics (and incorporating transcriptomics, proteomics and metabolomics) in combination, factory-specific detection and enumeration strategies can be developed and utilised. This will allow for a better understanding of the phage-host interactions occurring in these complex microbial communities and for more reliable and robust fermentation practices.

## DECLARATIONS

### Copyright

© The Author(s) 2022.
